# Bone Mineral Density and Body Composition are Associated with Circulating Angiogenic Factors in Post-menopausal Women

**DOI:** 10.1007/s00223-016-0186-7

**Published:** 2016-08-30

**Authors:** A. Spangenberg, N. Maghsoodi, D. Dulnoan, A. E. Moore, S. Edwards, M. L. Frost, G. Hampson

**Affiliations:** 1 Department of Clinical Chemistry and Metabolic Medicine, St Thomas’ Hospital, 5th Floor, North Wing, London, SE1 7EH UK; 2 Osteoporosis Unit, Guy’s Hospital, London, SE1 9RT UK; 3 Metabolic Bone Clinic, Department of Rheumatology, Guy’s Hospital, London, SE1 9RT UK

**Keywords:** Bone density, Body composition, Angiogenic factors

## Abstract

Lean mass (LM) and fat mass (FM) are closely related to bone mass (BM) in post-menopausal women, although their relative importance is unclear. Angiogenic factors which control angiogenesis may influence BM, LM and FM. The aim of the study was to compare the contribution of LM and FM to bone mineral density (BMD) and the association between these tissues and circulating angiogenic factors. The study population comprised of 392 post-menopausal women aged mean [SD] 61.8 [6.4] years. BMD was measured at the lumbar spine (LS), neck of femur and total hip (TH) by dual-energy X-ray absorptiometry (DXA). DXA scan was also used to determine LM and FM. Angiopoietin-1 and 2 (ANG-1, ANG-2) were measured by sandwich enzyme-linked immunosorbent assay. Following adjustment for confounders, significant positive independent associations were seen between LM with BMD at all skeletal sites (TH: *p* < 0.0001) and FM with BMD at the hip sites (TH: *p* = 0.004). When BMD and LM were regressed against the angiogenic factors, positive associations were seen between ANG-2 with LM (*p* = 0.002) and LS BMD (*p* = 0.05). Negative associations were observed between the ratio of ANG-1/ANG-2 with LS BMD (*p* = 0.014), TH BMD (*p* = 0.049) and LM (*p* = 0.029). FM and fat distribution (android/gynoid fat ratio) were negatively associated with ANG-1 (*p* = 0.006) and ANG-2 (*p* = 0.004), respectively. ANG-1 and ANG-2 may be involved in the maintenance of bone, muscle and fat mass.

## Introduction

Several cross-sectional studies have demonstrated significant associations between body composition and bone mass in post-menopausal women [1, 2]. Menopause has been known to be associated with an increased loss of bone mass, increased adipose mass and decreased muscle mass. Some studies report that lean mass may be a stronger predictor of BMD than fat mass, although the extent of the association may be age and sex dependent [3].

The association of lean mass with bone health is evident clinically in subjects with sarcopenia, defined as loss of muscle as a consequence of ageing. Sarcopenia has been linked with low bone mass, increased falls risk and fractures as a result of functional decline [4]. Fat mass has been associated with higher BMD and reduced odds ratio for low bone mass [5]. The association seems stronger in post-menopausal women, although the effect of fat mass may be related to its distribution as subcutaneous fat and visceral adipose tissue (VAT) may have differential effect on bone [6]. In a recent study, lean mass and fat mass have been shown to be independently associated with bone micro-architecture, although the relationship was different. Cortical area and thickness were significantly associated with lean mass whilst trabecular number and density were related to fat mass [7].

Adipose tissue, skeletal muscle cells or bone cells originate from the same pluripotent mesenchymal stem cells and may be regulated by similar factors [8]. The multiple cellular pathways connecting bone, skeletal muscle and fat cells are complex and remain unclear. Multiple factors are involved in the relationship between muscle and bone. Growth hormone and insulin-like growth factor-1 (IGF-1) have been shown to have positive effects on both and decline with ageing contributing to sarcopenia and/or osteoporosis [9]. The association between fat and bone has been linked to the local production of oestrogen by adipose tissue [10]. Other factors including cytokines and adipokines have also been implicated [11].

We hypothesise that angiogenesis or factors which regulate this process may be important in the maintenance of muscle, fat and bone tissue. Angiopoietin-1 (ANG-1) and 2 (ANG-2) together with vascular endothelial growth factor (VEGF) are important mediators of angiogenesis. ANG-1 and tyrosine kinase signalling are essential for regulating blood vessel development and the stability of mature vessels. ANG-2, however, works as an antagonist of ANG-1 and works with VEGF to facilitate cell proliferation and migration of endothelial cells. It functions as a vessel-destabilising molecule and sensitises the endothelial cells to the actions of exogenous cytokines or growth factors. The balance of these 2 angiopoietins is important in the regulation of angiogenesis [12]. Thus, the ratio between ANG-1 and ANG-2 is often used to investigate their effects.

It is known that angiogenesis plays an important role in the regulation of bone remodelling and growth [13]. Pro-angiogenic factors, such as vascular endothelial growth factor (VEGF) and angiopoietin (ANG), are expressed at skeletal remodelling sites supporting their role in vascular growth throughout skeletal tissue [14, 15]. In addition to their effect on vasculogenesis, they may also act directly on bone cells. VEGF has been shown to regulate osteoblasts survival and to act as a chemoattractant to osteoclasts [16]. Over-expression of ANG-1 leads to increase bone mass in transgenic mice [17]. Their release is stimulated by bone-derived cytokines as well as hypoxia, simulating bone injury. It has also been proposed that sarcopenia or muscle weakness due to ageing may be related to reduced capillary density in the muscles [18]. Levels of various angiogenic factors may decline in muscles with ageing. Therefore, the release of angiogenic factors in muscles could potentially contribute to the reversal of sarcopenia as previously suggested [19, 20]. These factors may exert positive effects on endothelial cells as well as myogenic progenitor cells locally or at distant sites when released in the circulation. Likewise the development and maintenance of adipose tissue throughout adult life are also closely dependent on the growth of a capillary network [21]. In addition, adipose tissue can initiate the formation of blood vessel as previously demonstrated, and in turn, endothelial cells in adipose tissue can promote the differentiation of pre-adipocyte presumably through the secretion of angiogenic factors which act directly on pre-adipocytes in a manner analogous to that seen in bone cells [22].

Thus, we postulate that angiogenic factors may be associated with bone, lean and fat mass and explain, in part, the mechanisms linking muscle, fat and bone. The aim of this study is to explore the relationship between these tissues and circulating angiogenic factors.

## Materials and Methods

### Study Population

This cross-sectional study comprised of 392 post-menopausal women aged mean [SD] 61.8 [6.4] years. The majority of participants (88.5 %) were recruited in the osteoporosis unit following referral for a DXA scan for measurement of their bone mineral density (BMD) or through community advertising for study volunteers. The rest (*n* = 45) were recruited when they attended the unit for a DXA scan prior to their visit to the metabolic bone clinic. Consecutive subjects were informed about the study and those who agreed to take part were entered in the study. Ethical approval was obtained from the St Thomas’ Hospital Research Ethics Committee. All participants gave informed consent. Bone mineral density (BMD) was measured at the lumbar spine (LS), neck of femur (FN) and total hip (TH). DXA scan was also used to determine body composition: lean and fat mass as well as visceral adipose tissue (VAT). Each study participant completed a questionnaire to capture their past and current medical and drug history. Fasting blood samples (overnight fast from 10.00 pm the previous day) were taken at the same visit and serum/plasma stored at −80 °C until analysis of the biomarkers. Exclusion criteria were history of metabolic bone diseases other than osteoporosis such as Paget’s disease, chronic kidney disease–mineral bone disorder (CKD-MBD) or vitamin D deficiency, endocrine/biochemical abnormalities such as thyroid disorders and primary hyperparathyroidism. None of the participants were on treatment with oestrogens, progestin, selective oestrogen receptor modulators, glucocorticoids or teriparatide. Ninety-one subjects were on current treatment with or had previously been on oral bisphosphonates. Participants demographics are summarised in Table 1.

**Table 1:** Summary of the demographics of the study population

Parameter	Mean ± SD (%)
Number of participants	392
Age (years)	61.8 ± 6.4
Years since menopause	12.5 ± 7.6
BMI (kg/m^2^)	25 ± 4.5
Previous fractures (%)	128 (32.6)
Current and past smoker (%)	135 (34.4)
Bone mineral density (BMD) g/cm^2^	
Lumbar spine (LS)	0.897 ± 0.12
Femoral neck (FN)	0.713 ± 0.104
Total hip (TH)	0.840 ± 0.109
Lean mass (kg)	40.1 ± 5.3
Fat mass (kg)	24.2 ± 8
Visceral adipose tissue (VAT) (kg)	0.43 ± 0.23
Android/gynoid ratio	0.87 ± 0.16

### Measurement of BMD and Body Composition by Dual-Energy X-ray Absorptiometry (DXA)

BMD was measured by DXA (Hologic QDR DXA Discovery scanner, Hologic, Inc., USA) on all study participants at the lumbar spine (LS), femoral neck (FN) and total hip (TH). Study participants were classified into three groups: those with normal BMD, osteopenia and osteoporosis according to the WHO criteria for diagnosing osteoporosis. The CV for BMD measurement using the spine phantom was 0.37 %.

Total lean mass (LM) and fat mass (FM) were also measured by DXA using whole-body software version 13.4.2:3. All subjects were positioned for the scans in accordance with manufacturer instructions. Percentage fat mass was derived from the proportion of total tissue mass made up of total fat mass. Quantification of fat mass in the android and gynoid regions fat mass was carried out as previously described [23]. The region of interest (ROI) for the assessment of android fat was defined with the caudal limit at the top of the iliac crest and the cephalic limit at the base of the skull. The height of the android ROI was automatically set to 20 % of the distance from the iliac crest to the base of the skull. The android ROI comprises of both VAT and subcutaneous adipose tissue (SAT). VAT is derived by subtracting SAT from the total android fat. The gynoid region includes the hips and upper thighs, and overlaps both the leg and trunk regions. The upper demarcation is below the top of the iliac crest at a distance of 1.5 times the android height. The total height of the gynoid region is two times the height of the android region. The results were expressed as the android/gynoid ratio. The CVs for the measurement of total lean and fat mass are 1 %. The VAT coefficient of variation is less than 10 %.

### Routine Biochemical Measurements

Routine laboratory tests including serum creatinine and albumin-corrected calcium were measured by standard laboratory methods using Roche analysers (Roche Diagnostics Limited, West Sussex RH15 9RY, UK). Estimated glomerular filtration rate (eGFR) was calculated using the MDRD formula [24]. Serum intact PTH was measured using Roche reagents on the Roche Elecsys 2010 analyser (Roche Diagnostics Limited). Inter- and intra-assay CVs were <5 % at mean concentrations of PTH of 41 and 105 ng/l, respectively.

### Measurement of Circulating Angiogenic Factors: VEGF, ANG-1 and ANG-2

VEGF, ANG-1 and ANG-2 concentrations were measured in serum by sandwich enzyme-linked immunosorbent assay (ELISA) technique (Duoset, R&D Systems Europe Ltd, Abingdon, UK) according to the manufacturers instructions. Different ELISAs were used which were specific for each factor. Standard curves were constructed using concentrations of VEGF, ANG-1 and ANG-2 ranging from 0–2, 0–10 to 0–6 ng/ml, respectively. The detection limit of the ANG-1, ANG-2 and VEGF assays was 0.15, 0.1 and 0.02 ng/ml, respectively. Inter-assay CV was 9.4 % at ANG-1 concentration of 45 ng/ml, 11 and 13 % at ANG-2 concentrations of 0.8 and 1.46 ng/ml, respectively. The inter-assay CV for VEGF was 10 % at VEGF concentrations of 0.26 ng/ml. The ratio of ANG-1 and ANG-2 was derived and included in the analyses.

### Statistical Analysis

Statistical analysis was performed using IBM SPSS Statistics 20. Mean and standard deviation (SD) were derived for all continuous variables. Multivariable linear regression was used to examine the associations between BMD at the LS, FN, TH with measures of body composition: LM, FM following adjustment for confounders: age, height, smoking habits, alcohol consumption, years since menopause, previous fractures and bisphosphonate treatment. Pearson correlation or Spearman’s rank correlation when data were non-normally distributed was used to explore the relationship between BMD at different sites, FM and LM with the angiogenic factors. Non-normal data were log-transformed. In separate multilinear regression models, the association between BMD, LM, FM, VAT, fat distribution, android/gynoid fat ratio with the angiogenic factors following correction for demographics and lifestyle variables was explored. Stepwise multilinear regression was also used to identify the strongest correlated variable. The independent variables collectively were linearly related to the dependent variable, as tested by plotting the studentised residuals against the unstandardised predicted values, and each independent variable was found to be linearly related to the dependent variable. Group comparisons were done using the unpaired Student’s ‘*t*’ test. A ‘*p*’ value of <0.05 (95 % confidence interval) was considered as statistically significant.

## Results

### Study Population

Three hundred and ninety-two post-menopausal women were studied. The study population’s demographics is summarised in Table 1. They were divided into three groups based on their BMD. Eighty-one women (20.7 %) had a BMD diagnosis of osteoporosis; ‘*T*’ score of <−2.5 at either the LS, FN or TH, 219 (55.9 %) had osteopenia; ‘*T*’ score between −1 and −2.5 and 92 (23.4 %) had normal BMD. One hundred and twenty-eight women (32.6 %) had previously sustained a fracture. Eighty-seven women (22 %) had previously been treated or were on oral bisphosphonates. Lean mass (LM), fat mass (FM), % FM and VAT in the whole study population were mean [SD] 40.15 [5.3] kg, 24.2 [8] kg, 35.7 [6.4] % and 0.43 [0.23] kg, respectively. The circulating concentrations of the angiogenic factors in the whole population were VEGF: 0.173[0.46] ng/ml, ANG-1: 39.9 [18.3] ng/ml, ANG-2: 0.914 [0.5] ng/ml. The concentrations of the angiogenic factors did not differ significantly between women on BPs compared to treatment-naïve participants. LM was significantly lower in subjects with osteoporosis compared to those with normal BMD (*p* < 0.001) or osteopenia (*p* < 0.001). There were significant differences in FM, VAT and % fat between the three groups. Subjects with osteoporosis had significantly lower FM (*p* < 0.001) and VAT (*p* = 0.001) compared to those with normal BMD. There was no significant difference in the angiogenic factors between subjects with osteoporosis, osteopenia or normal BMD. Data on BMD, body composition and circulating angiogenic factors are summarised in Table 2.

**Table 2:** Bone mineral density (BMD), body composition and biochemical parameters of post-menopausal women with normal BMD, osteopenia and osteoporosis

BMD, body composition and biochemical parameters	Normal BMD *N* = 92	Osteopenia *N* = 219	Osteoporosis *N* = 81
Bone mineral density (g/cm^2^)			
Lumbar spine (LS)	1.04 [0.07]	0.90 [0.09]**	0.76 [0.08]^##^
Femoral neck (FN)	0.84 [0.07]	0.70 [0.07]**	0.61 [0.06]^##^
Total hip (TH)	0.97 [0.07]	0.84 [0.08]**	0.73 [0.071]^##^
Body composition			
Lean mass (kg)	43.3 [6.3]	39.8 [4.3]**	37.5 [3.9]^##^
Fat mass (kg)	27.9 [9.5]	23.9 [7.0]**	21 [6.7]^##^
Visceral adipose tissue (VAT) (g)	493 [251]	422 [222]*	375 [216]^##^
Android/gynoid ratio	0.91 [0.14]	0.86 [0.16]*	0.82 [0.17]^##^
Biochemical parameters			
eGFR (ml/min)	78.9 [13.5]	79.9 [15]	79.2 [15]
Parathyroid hormone (PTH) (ng/l)	38.4 [12.2]	39 [11.5]	39.9 [10.6]
Serum albumin-corrected calcium (mmol/l)	2.38 [0.07]	2.37 [0.10]	2.36 [0.08]
Angiogenic factors			
Vascular endothelial growth factor (VEGF) (ng/ml)	0.141 [0.18]	0.17 [0.42]	0.213 [0.73]
Angiopoietin-1 (ANG-1) (ng/ml)	39.2 [19.5]	40.2 [17.7]	40 [19]
Angiopoietin-2 (ANG-2) (ng/ml)	0.95 [0.47]	0.91 [0.53]	0.89 [0.44]
ANG-1/ANG-2 ratio	46.9 [26.4]	54.9 [52]	50 [22.5]

* *p* < 0.05; ** *p* < 0.01 normal BMD v/s osteopenia, ^#^ *p* < 0.05; ^##^ *p* < 0.01 normal BMD v/s osteoporosis

### Relationship Between Fat Mass (FM), Lean Mass (LM) and Bone Mineral Density (BMD)

Following bivariate analyses, we observed significant positive correlation between FM and BMD at all skeletal sites LS (*r* = 0.256, *p* < 0.001), FN (*r* = 0.332, *p* < 0.001) and TH (*r* = 0.360, *p* < 0.001). Positive relationships were also seen between LM and BMD at all skeletal sites LS (*r* = 0.372, *p* < 0.001), FN (*r* = 0.376, *p* < 0.001) and TH (*r* = 0.419, *p* < 0.001). A positive correlation was seen between FM and LM (*r* = 0.625, *p* < 0.001). Following adjustment for confounders which included age, height, years since menopause, previous fractures, bisphosphonates therapy, smoking habits, alcohol intake, in a multivariable linear regression model, significant positive independent associations were seen between LM with BMD at all skeletal sites. Significant associations were seen between FM and BMD at the TH and FN only (Table 3). Using stepwise linear regression, LM was the strongest predictor of BMD at the LS, TH, FN (*p* < 0.0001) for each model composed of different sets of variables including FM.

**Table 3:** Linear regression analysis of bone mineral density (BMD) (g/cm^2^) as dependent variable with body composition: lean mass and fat mass (kg) following correction for covariates including age, years since menopause, height, fracture history, smoking habits, alcohol intake, bisphosphonate therapy

	Total hip (TH)			Femoral neck (FN)			Lumbar spine (LS)		
	Beta	*B* CI (95 %)	*p* value	Beta	*B* CI (95 %)	*p* value	Beta	*B* CI (95 %)	*p* value
Dependent variable: bone mineral density (g/cm^2^)									
Fat mass (kg)	**0.158**	**0.001, 0.004**	**0.004**	**0.169**	**0.001, 0.004**	**0.004**	0.062	−0.001, 0.003	0.312
Lean mass (kg)	**0.313**	**0.004, 0.009**	**0.000**	**0.221**	**0.002, 0.007**	**0.001**	**0.286**	**0.003, 0.010**	**0.000**

The label for standardised co-efficient is beta, and B refers to the unstandardised co-efficient. Significant variables are shown in bold

### Relationship Between BMD and the Angiogenic Factors: VEGF, ANG-1, ANG-2 and ANG-1/ANG-2 Ratio

BMD at the LS, FN, TH and WB was regressed against the angiogenic factors and ANG-1/ANG-2 ratio individually and adjusted for co-variates including age, years since menopause, smoking habits, alcohol intake, BMI, bisphosphonates use and biochemical variables, eGFR and PTH concentrations. A positive association was seen between LS BMD and ANG-2 (*p* = 0.055). Significant negative associations were observed between the ratio of ANG-1/ANG-2 with LS BMD (*p* = 0.014) and TH BMD (*p* = 0.049). No significant associations were observed between BMD at any site with VEGF (Table 4). Stepwise linear regression showed that, amongst the angiogenic factors, ANG-2 was the strongest predictor of LS BMD (*p* = 0.009) in a model which consisted of age, BMI, use of bisphosphonates, ANG-1 and ANG-2.

**Table 4:** Linear regression analysis of bone mineral density (BMD) as dependent variable with the angiogenic factors and ANG-1/ANG-2 ratio following correction for covariates including age, years since menopause, BMI, smoking habits, alcohol intake, bisphosphonate therapy, eGFR and PTH

	Total hip (TH)			Femoral neck (FN)			Lumbar spine (LS)		
	Beta	*B* CI (95 %)	*p* value	Beta	*B* CI (95 %)	*p* value	Beta	*B* CI (95 %)	*p* value
Bone mineral density (g/cm^2^)									
VEGF (ng/ml)	0.000	−0.020, 0.021	0.996	−0.015	−0.024, 0.017	0.745	−0.035	−0.034, 0.016	0.478
ANG-1 (ng/ml)	−0.048	−0.001, 0.000	0.297	−0.042	−0.001, 0.000	0.373	−0.080	−0.001, 0.000	0.102
ANG-2 (ng/ml)	0.046	−0.009, 0.029	0.305	0.001	−0.019, 0.019	0.978	**0.093**	**−0.001, 0.046**	**0.055**
ANG-1/ANG-2 ratio	**−0.089**	**−0.001, 0.000**	**0.049**	−0.028	0.000, 0.000	0.547	**−0.120**	**−0.001, 0.000**	**0.014**

The label for standardised co-efficient is beta, and *B* refers to the unstandardised co-efficient. Significant variables are shown in bold

### Relationship Between Lean Mass (LM) and the Angiogenic Factors: VEGF, ANG-1, ANG-2 and ANG-1/ANG-2 Ratio

There was a significant positive correlation between LM and ANG-2 (*r* = 0.12, *p* = 0.008) and a negative correlation with ANG-1/ANG-2 ratio (*r* = −0.11, *p* = 0.037). When LM was regressed against the angiogenic factors following adjustment for the demographics and lifestyle co-variates, the association with ANG-2 (*p* = 0.002) and ANG-1/ANG-2 ratio (*p* = 0.029) remained significant. We did not observe any significant association between LM and ANG-1 or VEGF (Table 5). Stepwise linear regression showed that ANG-2 was the strongest predictor of LM in a model which consisted of BMI and years since menopause (YSM) as variables.

**Table 5:** Linear regression analysis of body composition and android/gynoid ratio as dependent variable with the angiogenic factors and ANG-1/ANG-2 ratio following correction for covariates including age, years since menopause, BMI, smoking habits, alcohol intake, bisphosphonate therapy, eGFR and PTH

	Lean mass (kg)			Fat mass (kg)			Android/gynoid ratio		
	Beta	*B* CI (95 %)	*p* value	Beta	*B* CI (95 %)	*p* value	Beta	*B* CI (95 %)	*p* value
Body composition									
VEGF (ng/ml)	0.014	−0.737, 1.05	0.731	−0.005	−0.881, 0.708	0.831	0.063	−0.008, 0.050	0.153
ANG-1 (ng/ml)	0.004	−0.021, 0.024	0.922	**−0.064**	**−0.048, −0.008**	**0.006**	−0.015	−0.001, 0.001	0.741
ANG-2 (ng/ml)	**0.122**	**0.471, 2.077**	**0.002**	−0.043	−1.409, 0.035	0.062	**−0.123**	**−0.065, −0.012**	**0.005**
ANG-1/ANG-2 ratio	**−0.107**	**−0.036, −0.006**	**0.007**	0.007	−0.011, 0.016	0.762	0.036	0.000, 0.001	0.412

Significant variables are shown in bold. The label for standardised co-efficient is beta, and B refers to the unstandardised co-efficient

### Relationship Between Fat Mass (FM), VAT, % Body Fat and the Angiogenic Factors: VEGF, ANG-1, ANG-2 and ANG-1/ANG-2 Ratio

There were no significant correlation between total body FM and the angiogenic factors (ANG-1: *p* = 0.11, ANG-2: *p* = 0.17, VEGF: *p* = 0.25) following bivariate analyses. In contrast, we observed a significant positive correlation between VAT and VEGF (*r* = 0.11, *p* = 0.03) and a negative correlation between % body fat and ANG-2 (*r* = −0.116, *p* = 0.024). In multilinear regression analyses, there were negative associations between FM with ANG-1 (*p* = 0.004) and with ANG-2 (*p* = 0.046), although the association seemed closer with ANG-1. Statistically significant negative associations were shown between fat percentage and both ANG-1 (*r* = −0.108, *p* = 0.039) and ANG-2 (*r* = −0.184, *p* = 0.000). There were no significant associations following adjustment between VAT and the angiogenic factors. A significant negative association was observed between the android/gynoid ratio with ANG-2 (*p* = 0.004) (Table 5). Stepwise linear regression showed that ANG-1 was the strongest predictor of FM in a model (*p* = 0.005) which consisted of BMI as variable. Amongst the angiogenic factors, ANG-2 was the strongest predictor of the android/gynoid ratio in a model (*p* = 0.004) which also consisted of BMI.

## Discussion

In summary, our data show that although both LM and FM were positively associated with BMD in post-menopausal women, the relationship with LM was closer at all skeletal sites. Significant but contrasting associations were observed between ANG-1, ANG-2 and their ratio with BMD, LM and FM suggesting that these angiogenic factors may have a role in the regulation of bone mass and body composition.

The findings of the current study confirm the importance of LM and FM as determinants of BMD as previously described [1, 2]. Stronger positive associations have been reported between LM and BMD compared to FM in men and pre-menopausal women [25, 26]. In post-menopausal women, the effect of FM and LM on BMD has been shown to be similar [27]. This is in contrast to our findings as we showed that LM had a closer relationship with BMD compared to FM. Our data are in accordance with a previous study in a cohort of post-menopausal Vietnamese women [28]. Several hypotheses have been proposed to explain the significant association between body composition and BMD including the effects of endocrine, autocrine/paracrine and lifestyle factors including nutrition and physical activity [7]. We hypothesised that similar factors or mechanistic pathways may influence the metabolism of these tissues.

We thus investigated the role of angiogenic factors and their association with BMD, LM and FM. We observed an independent positive association between ANG-2 and BMD at the LS and negative associations between ANG-1/ANG-2 ratio with BMD at the LS and TH. Angiogenesis plays a key role in bone repair and remodelling on the endosteal surfaces of trabecular bone or the haversian system within cortical bone [13]. Bone remodelling not only involves bone resorption and formation but also a coupling with angiogenesis. ANG-1 and ANG-2 have opposing effects on the endothelial-specific receptor tyrosine kinase 2 (TIE2). ANG-1 stimulates TIE2 whilst ANG-2 is capable of antagonising this [12]. Thus, signalling through TIE2 depends on the balance between ANG-1 and ANG-2. The balance of these 2 factors in favour of ANG-2, as shown in our study, appears to indicate that new vessel formation may be associated with bone mass. Indeed, studies have shown a reduction in the number of capillaries density in histological samples of osteoporotic bones [29].

We also demonstrated similar associations between ANG-2 and ANG-1/ANG-2 ratio with LM. Our observations suggest that skeletal muscle capillarisation may play a role in the maintenance of muscle mass and a reduction in angiogenesis may contribute to sarcopenia and physical decline in elderly people [18]. Indeed, animal studies have shown that endothelial cell apoptosis and impairment in capillary function leads to a decline in muscle mass [30]. On the other hand, capillary proliferation results in improvement in muscle mass by presumably allowing oxygen and nutrient delivery to the muscle cells [20]. It has also been shown that aerobic exercise training can increase muscle capillarisation [31], although the effect of exercise on ANG-1 and ANG-2 is not known. All participants in the current study were ambulant; however, we do not have an accurate measure of their level of physical activity. Nevertheless, our findings, albeit cross-sectional in nature, are of interest although further confirmatory studies are required.

Adipose tissue is unique in its plasticity and undergoes expansion and regression throughout adult life [22]. The growth of adipose tissue requires the formation of a capillary network. It is associated with angiogenesis in a context-dependent manner [21]. Animal studies have shown that ANG-1 mRNA expression is downregulated in obesity and with weight change [32, 33]. ANG-1 expression is negatively related to adipose tissue weight [35], suggesting that ANG-1 may negatively affect adipose tissue growth and thus explaining, in part, the observed negative association between ANG-1 and FM in this study. Several other angiogenic factors including ANG-2 and the angiogenesis inhibitor endostatin are significantly elevated in either overweight or obese subjects compared to lean individuals [34]. It is thought, though as yet unproven, that adipose tissue itself may contribute to the elevated circulating levels of these factors. This was not, however, seen in our study. An explanation is that the expression of ANG-2 may depend on the anatomical distribution of fat tissue. A negative association between the android/gynoid ratio and circulating ANG-2 levels was observed, therefore suggesting higher ANG-2 to be associated with an android/gynoid ratio favouring a gynoid fat distribution. To our knowledge, there are no previous data showing such an association and this remains to be elucidated. Not only FM but fat distribution has been shown to contribute to bone strength in post-menopausal women. Indeed, a negative association between android/gynoid ratio and hip bone strength has previously been documented [35].

A major strength of the study is that we have carefully screened participants who are ambulant community-dwelling women in good health with no clinical evidence of cardiovascular disease or kidney disease. Another strength is that the cohort is well characterised which enabled us to adjust for confounders or covariates. However, there are some limitations to the study which should be taken into account when interpreting the findings. Firstly, because of the cross-sectional design of the study, we are not able to determine any causal relationship between the angiogenic factors with BMD, LM and FM. Another limitation is that we have assumed that circulating concentrations of these angiogenic factors reflect the local tissue concentrations. Although the participants did not have clinical cardiovascular disease at study entry, we are not able to exclude sub-clinical ischaemic disease which could affect the production and secretion of the angiogenic factors. Recruitment of some participants from the metabolic bone clinic may have introduced bias, although the number was relatively small. Finally, although all participants were reported as being ambulant, we did not have an accurate objective assessment of their physical activity level, a potential confounder, which can explain the association between BMD and LM. We did not also analyse other mediators which include oestradiol, cytokines, adipokines or growth factors such as IGF-1 or sclerostin [36] which may play a key role in the relationship between body composition and BMD. Sclerostin has been shown to be positively correlated with BMD and FM. In addition, we did not capture dietary or nutritional intake which is associated with bone health, FM as well as LM.

In conclusion, we have shown that ANG-1, ANG-2 and ANG-1/ANG-2 ratio display significant associations with components of body composition and bone mass. The differing associations identified with FM compared to LM and BMD may reflect the differential regulation of the angiopoietins by factors produced by adipose tissue such as adipokines. Our data therefore suggest that ANG-1 and ANG-2 may affect bone, muscle and fat mass. Longitudinal studies are required to establish a deeper understanding and causal relationship between bone, LM, FM and the angiogenic factors.
